# Xp;Yq Unbalanced Translocation with Pseudoautosomal Region Aberrations in a Natural Two-Generation Transmission

**DOI:** 10.1155/2020/4976204

**Published:** 2020-12-01

**Authors:** Yuting Jiang, Yang Yu, Han Zhang, Hongguo Zhang, Meiling Sun, Ruizhi Liu

**Affiliations:** ^1^Center for Reproductive Medicine, Center for Prenatal Diagnosis, First Hospital of Jilin University, Changchun, China; ^2^Jilin Engineering Research Center for Reproductive Medicine and Genetics, Changchun, China

## Abstract

Translocations involving X and Y chromosomes rarely occur in humans and may affect reproductive function. We investigated an Xp:Yq unbalanced translocation with pseudoautosomal region (PAR) aberrations in a natural two-generation transmission. We report the case of an azoospermic male and his fertile mother without any other abnormal clinical phenotypes, except for short stature. Cytogenetic methods, including karyotyping and fluorescence *in situ* hybridization (FISH), revealed the translocation. Chromosomal microarray comparative genomic hybridization (array-CGH) was used to investigate the regions of Xp partial deletion and Yq partial duplication. Final chromosome karyotypes in the peripheral blood of the infertile male and his mother were 46,Y,der(X)t(X;Y)(p22.33;q11.22) and 46,X,der(X)t(X;Y)(p22.33;q11.22), respectively. Short-stature-homeobox gene deletion was responsible for the short stature in both subjects. PAR aberrations and AZFc duplication may be a direct genetic risk factor for spermatogenesis. This report further supports the use of routine karyotype analysis, FISH-based technology, and array-CGH analysis to identify derivative chromosomes in a complex rearrangement.

## 1. Introduction

Approximately 15% of couples cannot conceive naturally, and deficiencies in males account for 50% of these cases [1, 2]. Spermatogenic failure is a significant cause, typified by azoospermia [3]. To date, approximately 20% of patients with azoospermia have had a genetic defect. The most frequent genetic causes of idiopathic nonobstructive azoospermia are numerical and structural abnormalities and azoospermia factor (AZF) microdeletions in the Y chromosome [4]. X and Y chromosome translocations rarely occur and may affect reproductive function and typically display breakpoints at Xp22 and Yq11 after cytogenetic analysis [5]. Previously, females with X:Y translocations usually had normal clinical characteristics, including intelligence and reproductive function, except for short stature [6]. In contrast, depending on deletions in the X chromosome, males with these karyotypes are reported as liveborn males with multiple congenital anomalies or even stillborn [7]. In azoospermic patients, X:Y translocation is rarely described.

The pseudoautosomal regions (PAR1 and PAR2) of human X and Y chromosomes undergo pairing and recombination during meiosis [8]. In human male spermatogenesis, X-Y chromosome pairing in PARs is believed to play an important role [9]. PAR1 deletions are associated with infertility; however, the function of most PAR genes is unclear. The short-stature-homeobox (*SHOX*) gene is the best-known gene in PAR1. Haploinsufficiency of the SHOX gene is associated with short stature [10]. Here, we present the clinical characteristics and results of cytogenetics, FISH, and array-CGH analysis of an azoospermic patient with unbalanced Xp:Yq translocation. We discuss X:Y chromosomal translocations in infertile men that produce rearrangement in PARs to understand the phenotype for the purpose of genetic counseling.

## 2. Materials and Methods

### 2.1. Patients

A 25-year-old male was referred to our Center for Reproductive Medicine, First Hospital of Jilin University, owing to primary infertility persisting for 2 years. No remarkable risk factors for infertility were identified in his medical history. Further, no abnormal characteristics were revealed after objective physical examination performed by an andrologist, except for short stature of 165 cm. Northern Han Chinese males have an average height of 172.5 ± 5 cm [11]. Both side testicular volumes were 15 mL. Three routine semen analyses, according to the World Health Organization guidelines (5th edition), revealed no spermatozoa [12].

The patient's parents were aged 23 (mother) and 25 (father) years and 146 cm and 175 cm tall, respectively. The family history was unremarkable. This study was approved by the Ethics Committee of the First Hospital of Jilin University, and all patients provided informed consent to participate in the study.

### 2.2. Cytogenetic Analysis

The patient was referred for medical genetic evaluation, and a cytogenetic analysis was requested. Karyotyping of peripheral blood samples was performed on family members using standard G-banding. The diagnosis was made according to the International System for Human Cytogenetic Nomenclature 2016 (ISCN-2016).

### 2.3. Chromosomal Microarray Analysis (CMA)

An array-CGH analysis was used to identify the derivative X chromosome for further study. Microarray analysis was performed using the Affymetrix CytoScan 750K Array chip, in accordance with the manufacturer's protocol. The specific procedure followed has been previously reported [13]. Ensemble Resources (DECIPHER), a database of genomic variants, and Online Mendelian Inheritance in Man (OMIM) were used to analyze the chromosomal imbalance and clinical phenotype in this study.

### 2.4. FISH Procedure

Additionally, fluorescence *in situ* hybridization (FISH) testing was performed using Cytocell probes, as per the manufacturer's protocol (Cytocell Technologies, Cambridge, UK). Commercial probes were as follows: centromere region of the X chromosome (DXZ1, X-cen alphasatellite, spectrum aqua), *SRY* probe (spectrum red), and heterochromatin region of the Y chromosome q arm (DYZ1, Yq12 satellite III, spectrum green). Detailed experimental procedures were performed as described by An et al. [14].

## 3. Results

**Figure 1:**
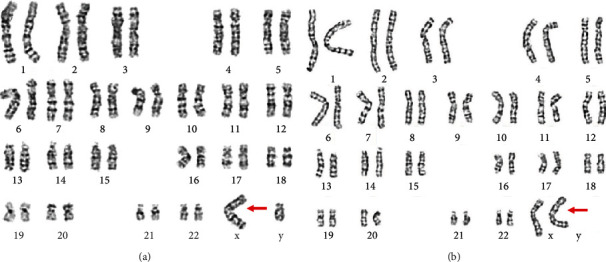
Karyotype analysis of the proband (a) and the mother of the proband (b) identified by GTG banding technique.

**Figure 2:**
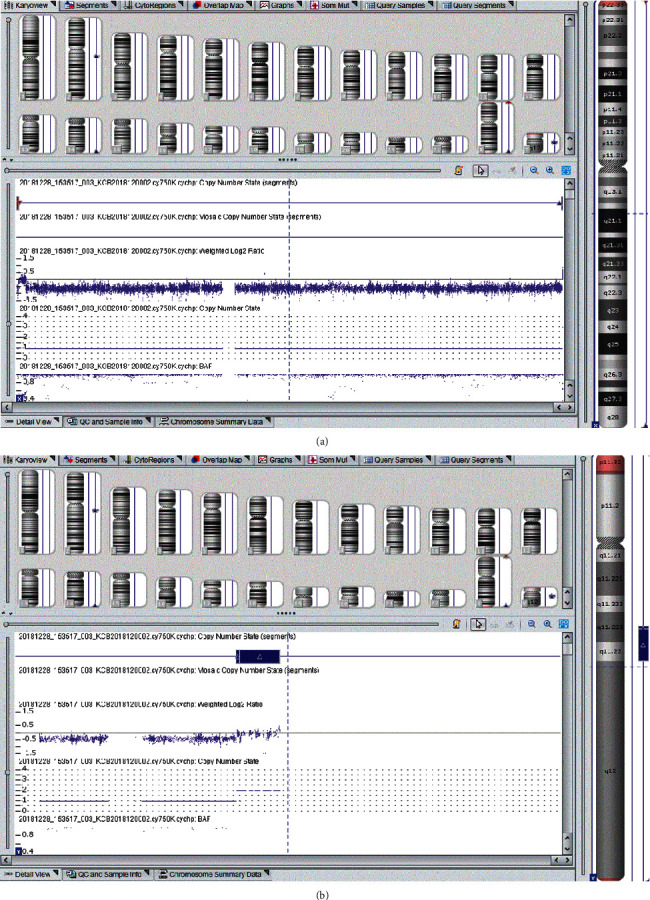
Array-CGH showing 0.55 Mb loss of Xp22.33 (a) and 4.283 Mb gain of Yq11.223-q11.23 (b) of the proband.

**Figure 3:**
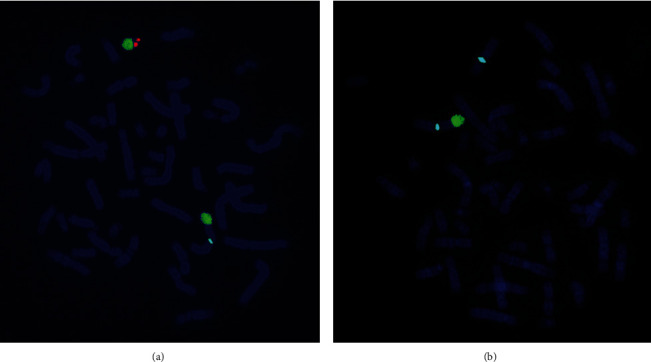
Metaphase-FISH results of the proband (a) and the mother of the proband (b) using three-color FISH with DNA probe specific for DXZ1 (spectrum aqua), *SRY* (spectrum red), DYZ1 (spectrum green), and DAPI (4′,6-diamidino-2-phenylindole; spectrum blue): *SRY* signal and heterochromatin region of the q arm of chromosome Y were showing the derivative X chromosome.

**Figure 4:**
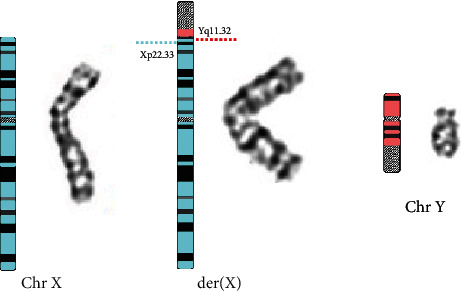
The ideogram of Xp;Yq unbalanced translocation. Y chromosome band nomenclature follows ISCN (2016).

The chromosomal karyotypic analysis showed an unbalanced translocation, comprising an extrachromosomal segment of unknown origin attached to the p arm of chromosome X. As initially reported, the karyotype was described as 46,Y,der(X)t(X;?)(p22.3::?) (Figure 1(a)); however, the exact breakpoint of the X chromosome was unclear. The proband's mother had the same derivative metacentric X (Figure 1(b)) as her son. The proband's father had a normal karyotype. CMA was applied to identify the derivative X chromosome for characterization. An abnormal molecular karyotype was determined as arr[GRCh37] Yq11.223q11.23 (24516978-28799654)x2 and arr[GRCh37] Xp22.33 (168551-719762)x0 (terminal 4.283 Mb gain of Yq11.223-q11.23 and terminal 0.55 Mb loss of Xp22.33) (Figure 2). The Xp22.33 deletion region contains 4 OMIM genes: *SHOX*, *PLCXD1*, *GTPBP6*, and *PPP2R3B.* The Y chromosome duplication contains the AZFc region (10 OMIM genes: *TTTY17A*, *TTTY4*, *DAZ1*, *CDY1*, *CSPG4P1Y*, *GOLGA2P2Y*, *BPY2*, *DAZ3*, *DAZ2*, and *TTTY3*) and PAR2 region (4 OMIM genes: *SPRY3*, *VAMP7*, *IL9R*, and *WASH6P*) (Table 1). Subsequently, FISH probes were used for further verification of the derivative X chromosome for both patient and mother. FISH, using a centromere probe specific for chromosomes X, SRY gene signal, and heterochromatin region of the q arm of chromosome Y, showed one X centromeric probe signal and one DYZ1 probe signal, but no *SRY* probe signal on the derivative X chromosome in either case (Figure 3). We speculate that the karyotype of the derivative X chromosome was der(X)(Yqter→Yq11.223::Xp22.33→Xqter). The ideogram of Xp:Yq unbalanced translocation is shown in Figure 4.

## 4. Discussion

Here, we report an azoospermic and short stature male with an inherited Xp:Yq unbalanced translocation, characterized by FISH and array-CGH analysis for exact karyotype interpretation. At least 14 males with Xp22:Yq11 translocations have been reported in the literature (Table 2). Males with these karyotypes are reported to have multiple congenital anomalies. Although most affected males display short stature, other significant phenotypic findings are commonly present, including strabismus and flat nasal bridge, ichthyosis, mild mental retardation, chondrodysplasia punctata, epilepsy, and cryptorchism. Females with X:Y translocations are typically short with normal intelligence and normal reproductive function, but some individuals may exhibit mild intellectual disability [6]. In our cases, the proband's mother showed a short stature (146 cm) but no other abnormal clinical characteristics. Theoretically, a female with a 46,X,der(X)t(X;Y) karyotype should have normal reproductive function and be able to produce normal oocytes [15, 16]. The number of der(X)t(X;Y) gametes and normal X chromosome gametes would be greater than the abnormal gamete types, which would not cross the checkpoint in the cell cycle. Therefore, the most common embryo types that der(X)t(X:Y) females and normal males have would be der(X)t(X:Y), der(Y)t(X:Y), and normal karyotype [16].

**Figure 5:**
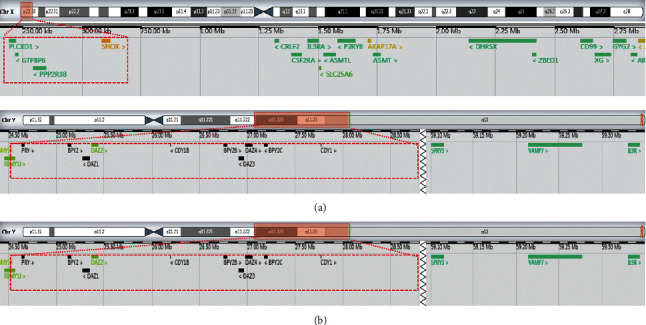
Scale representation of the deletion region in the distal short arm of chromosome X and the duplication region in the distal long arm of chromosome Y (https://decipher.sanger.ac.uk/).

In our case, the derivative X chromosome comprised a partial deletion of PAR1 on Xp with replacement with the duplicated segment from the q arm of the Y chromosome (q11.22→qter). X and Y chromosomes exhibit two pseudoautosomal regions, with synapsis and recombination during the prophase of the first meiotic division. PAR1 (approximate length 2.6 Mb) is located at Xp22.3 and Yp11.32, and PAR2 (approximate length 320 kb) is located at Xq28 and Yq12 [17]. To date [8], 24 and four genes have been discovered in the PAR1 and PAR2 regions, respectively. However, the function of most PAR genes is unknown. A previous study suggests that polymorphism genes in PAR are associated with mental disorders [18]. PAR genes in the DECIPHER database were reviewed. Copy number variations (CNVs) are in italics. Loss of CNVs in PAR1 genes and gain of CNVs in PAR2 genes are in bold and underlined, respectively (Table 1). The PAR1 deletion in our case contains four OMIM genes: *SHOX* (OMIM:312865), *PLCXD1* (OMIM: 300974), *GTPBP6* (OMIM: 300124), and *PPP2R3B* (OMIM: 300339) (Figure 5). Haploinsufficiency of the SHOX gene is associated with short stature and diverse skeletal anomalies, such as Leri-Weill dyschondrosteosis (LWD) [19]. *PLCXD1* is a pseudoautosomal gene that encodes an enzyme belonging to a subfamily of PIPLCs. This enzyme includes only the catalytic X domain for controlling many cellular processes by regulating cytosolic calcium and/or the activity of protein kinases [20]. *GTPBP6* is also known as pseudoautosomal GTP-binding protein-like, and, similar to other pseudoautosomal genes, it escapes X inactivation and has a functional homolog on the Y chromosome [21]. Yan et al. demonstrated that forced overexpression of the regulatory subunit of *PPP2R3B* perturbs cell cycle progression, causing cell cycle arrest in the G1 phase [22]. Moreover, CMA detected a PAR2 duplication with four genes: *SPRY3* (OMIM:300531), *VAMP7* (OMIM: 300053), *IL9R* (OMIM: 300007), and *WASH6P*. Active X and Y alleles of *SPRY3* showed delayed replication relative to an active X allele during the cell cycle [23]. An increased copy number of *VAMP7* can disrupt human male urogenital development through altered estrogen action [24]. Deletion of *IL9R* or other adjacent loci in the long-arm pseudoautosomal region might be responsible for some phenotypic features associated with Yq deletions, such as short stature, azoospermia, learning disabilities, and facial dysmorphism [25]. The function of the WASH6P gene remains unclear. The CNVs in the PAR genes lead to an extraordinarily high recombination frequency of sex chromosomes. However, the pairing of sex chromosomes ensures accurate segregation of chromosomes during spermatogenesis.

The AZF region is predisposed to structural rearrangement owing to numerous ampliconic and palindromic sequences [26]. Our cases show the extra duplication of the AZFc and heterochromatin regions of the Y chromosome in both the proband and mother. The AZFc duplication region contains 10 OMIM genes, including *TTTY17A*, *TTTY4*, *DAZ1*, *CDY1*, *CSPG4P1Y*, *GOLGA2P2Y*, *BPY2*, *DAZ3*, *DAZ2*, and *TTTY3* (Figure 5). In general, the hidden Y chromosome fragment is rarely found in females with normal fertility. However, our patient had normal clinical characteristics, with the exception of short stature, and normal fertility. AZFc region duplications with an increased DAZ copy number are a risk factor for dysfunction in spermatogenesis [27, 28]. In addition, stability of the Y chromosome is important for the progression of spermatogenesis. Gene disruption or positional effects caused by translocations may also lead to arrested spermatogenesis during the meiotic prophase.

In conclusion, here, we report an Xp:Yq unbalanced translocation with PAR aberrations in a natural two-generation transmission. PAR aberrations and AZFc duplication may be a direct genetic risk factor for dysfunctional spermatogenesis. This report further supports the use of routine karyotype analysis, FISH-based technology, and array-CGH analysis to identify derivative chromosomes in a complex rearrangement.

## Figures and Tables

**Table 1:** PAR genes from the DECIPHER database. Copy number variations (CNVs) referenced were in italics. The loss of PAR1 gene (*PLCXD1*, *GTPBP6*, *PPP2R3B*, and *SHOX*) CNVs was in bold. The gain of PAR2 gene (*SPRY3*, *VAMP7*, *IL9R*, and *WASH6P*) CNVs was underlined.

Pseudoautosomal region	Gene	Gene symbol	Normal male	Proband
PAR1	*PLCXD1*	Phospatidylinositol-specific phospholipase C,X domain containing 1	*2×*	**1×**
	*GTPBP6*	GTP binding protein 6	*2×*	**1×**
	*PPP2R3B*	Protein phosphatase 2, regulatory subunit B	*2×*	**1×**
	*SHOX*	Short stature homeobox	*2×*	**1×**
	*CRLF2*	Cytokine receptor-like factor 2	*2×*	*2×*
	*CSF2RA*	Colony-stimulating factor 2 receptor, alpha	*2×*	*2×*
	*IL3RA*	Interleukin 3 receptor, alpha	*2×*	*2×*
	*SLC25A6*	Solute carrier family 25, member A6	*2×*	*2×*
	*ASMTL*	Acetylserotonin omethyltransferase-like	*2×*	*2×*
	*P2RY8*	Purinergic receptor P2Y, G-protein coupled, 8	*2×*	*2×*
	*AKAP17A*	A-kinase anchoring protein 17A	*2×*	*2×*
	*ASMT*	Acetylserotonin O-methyltransferase	*2×*	*2×*
	*DHRSXY*	Dehydrogenase/reductase (SDR family) X-linked	*2×*	*2×*
	*ZBED1*	Zinc finger, BED-type containing 1	*2×*	*2×*
	*CD99*	CD99 molecule	*2×*	*2×*
	*XG*	XG blood group	*2×*	*2×*
PAR2	*SPRY3*	Sprouty RTK signaling antagonist 3	*2×*	3×
	*VAMP7*	Vesicle associated membrane protein 7	*2×*	3×
	*IL9R*	Interleukin 9 receptor	*2×*	3×
	*WASH6P*	WASP family homolog 6, pseudogene	*2×*	3×

**Table 2:** Reported males with der(X)t(Xp;Yq) translocations.

Refs.	No.	Karyotype	Origin	Age (years)	Semen analysis	Height (cm)	L/R testis volume (mL)	Other abnormalities
Present study		46,Y,der(X)t(X;Y)(p22.33;q11.22)	Maternal	25	Azoospermia	165	15/15	Normal phenotype
Cheng et al. [6]	1	46,Y,der(X)t(X;Y)(p22;q11.2)	Maternal	1	NP	70.5 cm (<2 SD)	Cryptorchism	Mental retardation and developmental delay, ichthyosis, concealed penis, a small scrotum, an undescended testis in his left lateral inguinal region, and a small testis in the right lateral scrotum.
Bukvic et al. [15]	2	46,Y,der(X),t(X;Y)(p22;q12)	NP	3	NP	88 cm tall (<5th centile)	NP	Brachycephaly, flattened nasal bridge, convergent strabismus, dry and scaly skin, global motor delay, and hyperactive behavior.
Doherty et al. [29]	3	46,Y,der(X)t(X;Y)(p22.3; q11.2).ish der(X) (DXZ1+, KAL+, STS-, SHOX-)	Maternal	24	NP	163	NP	Epilepsy, X-linked ichthyosis, Madelung deformities, mesomelia, normal intelligence, and attention deficits.
	4	46,Y,der(X)t(X;Y)(p22.3; q11.2).ish der(X) (DXZ1+, KAL+, STS-, SHOX-)	Maternal	23	NP	157	NP	Epilepsy, X-linked ichthyosis, Madelung deformities, mesomelia, normal intelligence, and attention deficits.
Ha et al. [30]	5	46,Y,der(X)t(X;Y)(p22.3;q11.2)	Maternal	3	NP	NP	Cryptorchism	Hypertelorism, clinodactyly, ear anomaly, simian crease, renal anomalies, and mild mental retardation.
Yen et al. [5]	6	46,Y,t(X;Y)(p22.3;q11.2)	Maternal	13 months	NP	NP	NP	Severe developmental delay, seizures, and ichthyosis.
Gabriel-Robez et al. [31]	7	46,Y,der(X),t(X;Y)(p22.3;q11)	NP	32	Azoospermia	Average stature	NP	Psychological disturbances.
	8	46,Y,der(X),t(X;Y)(p22.3;q11)	Maternal	40	Azoospermia	Average stature	NP	Psychological instability
Ballabio et al. [32]	9	46,Y,der(X)t(X;Y)(p22;q11)	Maternal	6	NP	105 cm (<3SD)	NP	Flat nasal bridge, chondrodysplasia punctata, ichthyosis, severe mental retardation, and epilepsy.
	10	46,Y,der(X)t(X;Y)(p22;q11)	Maternal	6	NP	113 cm, 25th centile	NP	Antimongoloid slant, strabismus and flat nasal bridge, ichthyosis, mild mental retardation, and chondrodysplasia punctata.
Ohdo et al. [33]	11	46,Y,der(X),t(X;Y)(p22.3;q11.1)	Maternal	Infant	NP	45.3 cm (<3SD)	NP	Peculiar face, congenital heart disease, dry and scaly skin, and growth and psychomotor retardation.
Ross [34]	12	46,Y,der(X),t(X;Y)(p22.3;q11)	Maternal	19	NP	<3SD	NP	Ichthyosis.
Metaxotou et al. [35]	13	46,Y, der(X),t(X;;)(p22;q11)	Maternal	14	NP	146	8/10	Hypogonadism, ichthyosis, and mental retardation.
Tiepolo et al. [36]	14	46,Y,t(X;Y)(Xqter→Xp22.2: :Yq11→Yqter)	Maternal	4 months	NP	NP	Cryptorchism	Flattened nasal bridge, convergent strabismus, bilateral simian creases, and small penis.

NP: not reported; SD: standard deviation.

## Data Availability

The data used to support the findings of this study are included within the article.
